# Children and young people’s mental health in England, Scotland and Wales – case studies using linked data from the Networked Data Lab.

**DOI:** 10.23889/ijpds.v7i3.1866

**Published:** 2022-08-25

**Authors:** Sebastien Peytrignet, Fiona Grimm

**Affiliations:** 1 The Health Foundation

## Objectives

The Networked Data Lab is a UK-wide network of analytical teams embedded in the health and care system . In 2021-22, we used linked data to answer questions on children and young people’s (CYP) mental health – a key issue in the NHS agenda, due to the rising prevalence of these conditions among CYP and the difficulty in accessing services.

## Approach

To enable novel analyses, each of our five partners linked new data sources to existing routine health and care data, e.g. from the Mental Health Services Dataset (England), Welsh Ambulance Services Trust (Wales) and Child and Adolescent Mental Health Services (Scotland). After linking in these datasets to their data models, we followed a federated analysis approach where each partner answered questions that were specifically relevant to their local stakeholders. Our overarching theme was inequalities in access to services, with sub-analyses focussing on issues such as the role of the ambulance service in crises, the transition from children to adult services and drug prescription patterns among young people.

## Results

A previous NHS England survey showed that mental health conditions have become more common among of 6-16 year olds, rising from one in nine young people with a probable condition in 2017, to one in six in 2021. Synthesising findings from across our five partners, we will present new analysis into patterns of mental health service use and crisis care, including variation by demographic characteristics such as age, sex and socioeconomic deprivation. We will also present new findings on rejected referrals, the role of the ambulance service in crisis care and retention during the transition from CAMHS to adult services.

## Conclusion and Relevance

This collaboration between five partners (each embedded in their local health systems as commissioners and researchers) showed that data linkages on children and young people’s mental health can provide unique insights that local service planners and national policymakers can act on.

